# Frequency-Domain Multiplexing Readout with a Self-Trigger System for Pulse Signals from Kinetic Inductance Detectors

**DOI:** 10.1007/s10909-018-1911-6

**Published:** 2018-04-16

**Authors:** Y. Yamada, H. Ishino, A. Kibayashi, Y. Kida, N. Hidehira, K. Komatsu, M. Hazumi, N. Sato, K. Sakai, H. Yamamori, F. Hirayama, S. Kohjiro

**Affiliations:** 1 Department of Physics, Okayama University, 3-1-1 Tsushimanaka, Kita-ku, Okayama Japan; 2 KEK, High Energy Accelerator Research Organization, 1-1 Oho, Tsukuba, Ibaraki 305-0801 Japan; 3 NASA Goddard Space Flight Center, Greenbelt, MD 20771 USA; 4 CRESST II - University of Maryland, Baltimore County, MD 21250 USA; 5 AIST, National Institute of Advanced Industrial Science and Technology, 1-1-1 Umezono, Tsukuba, Ibaraki 305-8560 Japan

**Keywords:** Kinetic inductance detectors, Frequency-domain multiplexing readout, Self-trigger system, Phonon detection

## Abstract

We present the development of a frequency-domain multiplexing readout of kinetic inductance detectors (KIDs) for pulse signals with a self-trigger system. The KIDs consist of an array of superconducting resonators that have different resonant frequencies individually, allowing us to read out multiple channels in the frequency domain with a single wire using a microwave-frequency comb. The energy deposited to the resonators break Cooper pairs, changing the kinetic inductance and, hence, the amplitude and the phase of the probing microwaves. For some applications such as X-ray detections, the deposited energy is detected as a pulse signal shaped by the time constants of the quasiparticle lifetime, the resonator quality factor, and the ballistic phonon lifetime in the substrate, ranging from microseconds to milliseconds. A readout system commonly used converts the frequency-domain data to the time-domain data. For the short pulse signals, the data rate may exceed the data transfer bandwidth, as the short time constant pulses require us to have a high sampling rate. In order to overcome this circumstance, we have developed a KID readout system that contains a self-trigger system to extract relevant signal data and reduces the total data rate with a commercial off-the-shelf FPGA board. We have demonstrated that the system can read out pulse signals of 15 resonators simultaneously with about 10 Hz event rate by irradiating $\alpha$ particles from $^{241}$Am to the silicon substrate on whose surface aluminum KID resonators are formed.

## Introduction

Popularity of large detector arrays containing thousands of superconducting detectors has been increasing in recent years. In such applications, it is critical to lower the number of readout cables to reduce the heat loading from room temperature to the sub-Kelvin temperature at which superconducting detectors operate. Kinetic inductance detectors (KIDs) [1, 2], a type of superconducting detectors, are promising in this regard for use at low temperatures. A single transmission feed line is inductively or capacitively coupled to microwave resonators of different frequencies. The probe microwaves in the transmission line flow into each corresponding resonator. When external energy in photons or phonons is deposited into a superconducting resonator, Cooper pairs are broken and quasiparticles are created. The increase in quasiparticles raises kinetic inductance and lowers the corresponding resonant frequency. As a result, the amplitude and the phase of the probing microwaves going through the feed line are changed. Photons or phonons can be detected by measuring this change in the signal; this is the detection principle of KIDs. Using this detection method, frequency-domain multiplexing readout of the multiple resonant frequencies is possible, allowing us to read out multiple channels with a single wire.

Specification requirements to the multiplexed readout system for measurements with KID arrays vary depending on the applications. Some experiments use existing programmable digital logic circuits, such as field-programmable gate arrays (FPGAs), whose specifications are easily changed. To name a KID readout system using FPGA, NIKEL_AMC developed for NIKA2 is the latest [3]. This system is used for the submillimeter wave astronomy. ARCONS [4, 5] employs a multiplexed readout system using Reconfigurable Open Architecture Computing Hardware (ROACH) [6, 7]. Both systems have developed dedicated hardware to read out KIDs with high bandwidths.

We introduce here a system we have developed to simultaneously read out multiple channels from KIDs using a FPGA board available commercially. We have developed such a system to read out 32 resonators for 100 Hz sampling rate, aiming for the observation of the submillimeter astronomy or the cosmic microwave background [8]. In order to increase the data bandwidth for a readout of pulse signals with a few $\upmu$s width, we have extended our previous readout system by implementing a self-trigger system which extracts relevant pulse signals and hence effectively reduces the total data rate based on a technique in [9]. This apparatus has an advantage of being relatively low cost, and the modification was rather simple due to the use of commercial Xilinx FPGA board, allowing us to have a scalability to enlarge the system using multiple boards for the readout of thousands of channels.

In this paper, we report on the development of a readout system of KID pulse signals with a self-trigger system. We first explain the readout electronics we have used, the analog circuit and digital FPGA system. Then, we will give demonstrative results using our system to simultaneously read out 15 pulse signals.

## Readout Electronics

The readout system we have developed is based on a commercially available Kintex-7 DSP Kit from Xilinx. It consists of a high-speed analog daughter board (FMC150) mounted on a digital mother board (KC705) connected with a Low-Pin Count (LPC) connector. The former is equipped with two channels, each of ADC and DAC for signal interface, and the latter utilizes Kintex-7 FPGA for processing the signals for our purpose. Our readout system makes use of the IQ mixing technique.

**Fig. 1:**
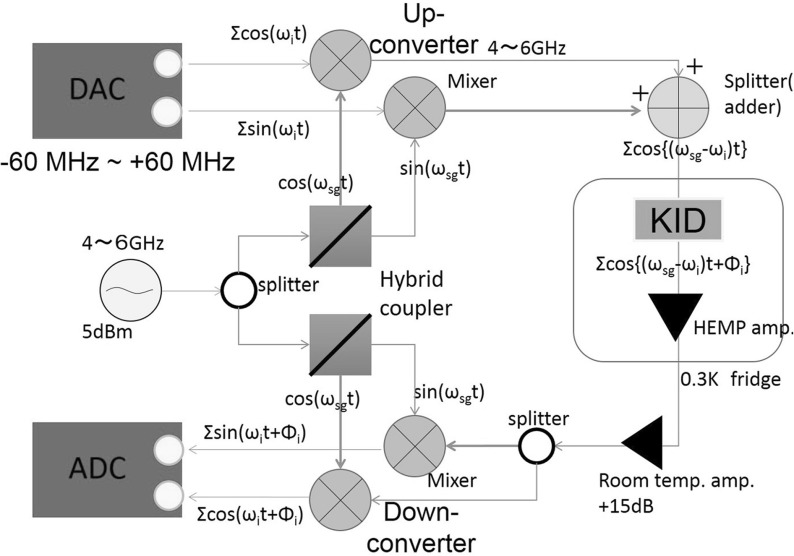
Overview of the analog system. Details of this are explained in the text (Color figure online)

Figure 1 shows an overview of the analog system. A comb of frequencies is generated in the frequency range from $-\,60$ to $+\,60$ MHz, where the negative frequency waves have the phase difference of $\pi$ from the positive-frequency waves. The two outputs from the DAC, having a relative phase difference of $\pi /2$, are up-converted to a frequency of 4–6 GHz to match the resonant frequencies in KIDs using a Local Oscillator (LO), which also produces two microwaves with a phase difference of $\pi /2$, and then are combined into a single wave to be fed to the KID in the cryostat. The output microwaves from the KID are amplified by a HEMT cryogenic amplifier with a 30 dB gain and are further amplified by a room-temperature amplifier. The microwaves are split into two, and the two waves are down-converted with the LO microwaves with $\pi /2$ phase difference and are fed into the ADC. The ADC has a resolution of 14 bits with the input range of 2 V peak to peak and operates at a sampling rate of 250 MSPS. The DAC has a resolution of 16 bits with 1 V peak to peak at 800 MSPS. We set our bandwidth up to a half of the Nyquist frequency and set the frequency range of $-\,60$ to $+\,60$ MHz.

**Fig. 2:**
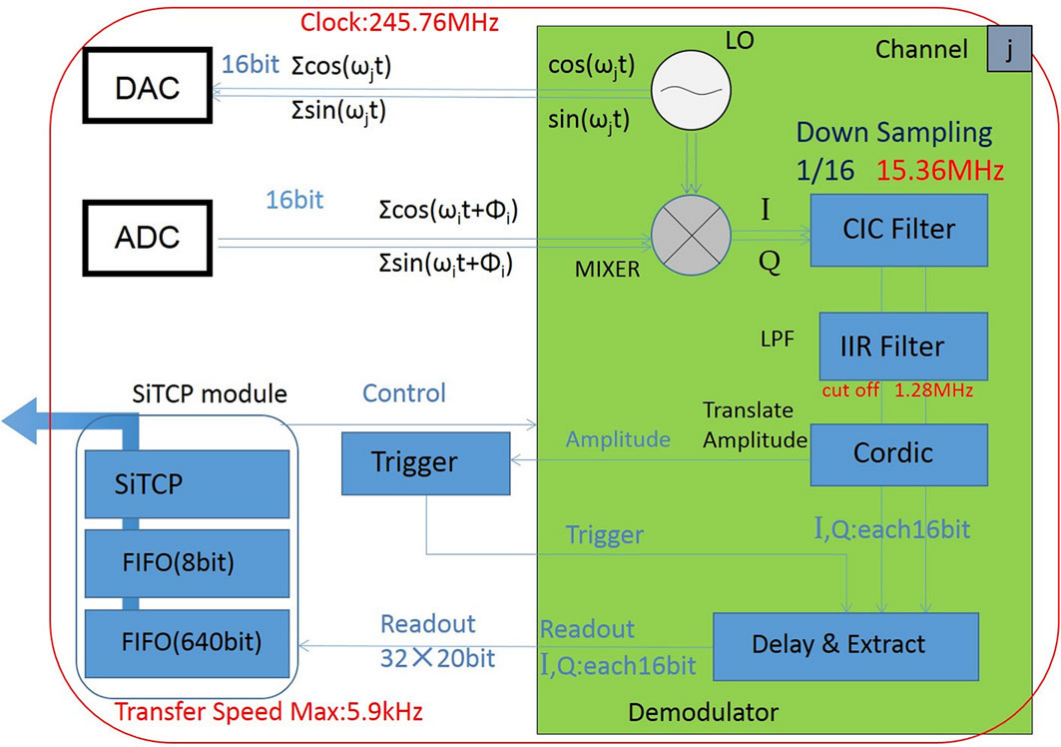
Overview of the digital system implemented as the logic in firmware in the Kintex-7 FPGA chip (Color figure online)

Figure 2 shows an overview of the digital circuit implemented in the FPGA chips. The firmware for the system is developed in VHDL. We create microwaves in a frequency comb using a direct digital synthesizer (DDS). The frequencies are separated by about 6 MHz to reduce the cross talks in the frequency domain, as the resonators in KIDs we have developed have the minimum time constant $\tau$ of about 500 ns, corresponding to 2 MHz bandwidth, that is determined by the resonator quality factor *Q*; $\tau =Q/\pi f_0$, where $f_0$ is the resonant frequency. Hence, the maximum number of frequencies we can use with our KID is 20. The actual KID does not have uniform frequency separations, and the resonant frequencies are displaced by 1–2 MHz from the designed value. We set the microwave frequencies by hand by measuring the values using a vector network analyzer. The LO frequency in the analog system is also tuned so that the up-converted frequency comb covers the actual KID’s resonator frequencies. The microwaves measured by ADC are fed into a mixer to create in-phase/quadrature signals. The *I*/*Q* signals go through low-pass filters, cascaded integrator comb (CIC) filter, and infinite impulse response (IIR) filter, reducing the bandwidth to 1.28 MHz. Further, the amplitude of the *I* / *Q* signals ($=\sqrt{I^2+Q^2}$) is calculated by COordinate Rotational DIgital Computer (CORDIC) to be used as a trigger signal to extract pulse signals. Trigger function is implemented within the logic circuit so that no external trigger signal is necessary, i.e., only *I* and *Q* signals that carry amplitude and phase information are read out. When the sum of the amplitude in the trigger system exceeds a threshold value we set, the extract module starts to draw out the *I* / *Q* signals. Extraction timing of the *I* / *Q* signals is tuned by applying a delay so that the pulse signals fall within the start and stop timings of the extraction module. The extracted length of data is $\sim \,33~\upmu$s for 512 samples, two of which are reserved for a header and a footer to be added by the extract module for data information such as DAQ time. The data read out are then transferred to a PC by TCP and UDP protocols implemented on a SiTCP [10] module for storage. Various slow controls are also possible using SiTCP technique; they include trigger mode (random or self-trigger), frequency setting of individual LOs, start and stop of the readout equipment, reset to clear the FIFO data, setting of the delay time to start reading data, or trigger level setting.

## Demonstration of KID Multiplexed Readout

**Fig. 3:**
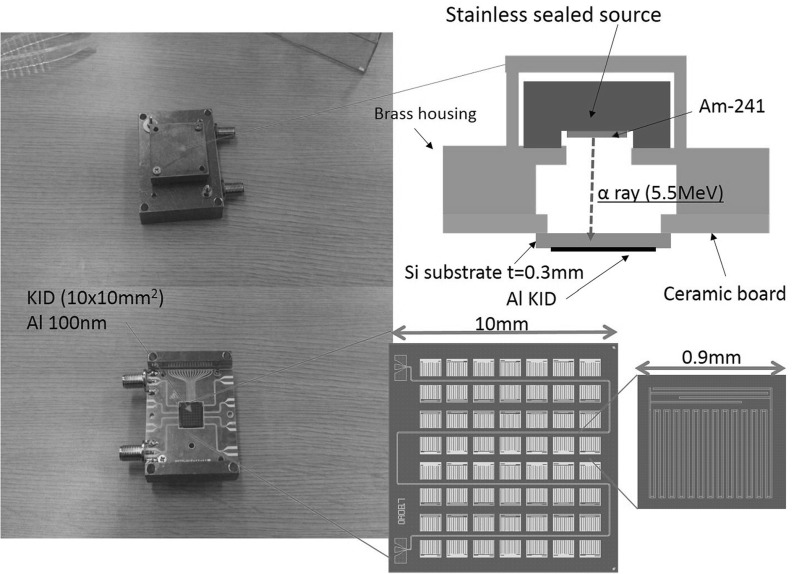
Design and setup of the KID used for the phonon signal measurement with our readout system. Top-left picture shows the brass housing in which a sealed source is placed, and the left bottom shows the ceramic board that places the KID chip in its center. Right top depicts the arrangement of the source and the KID inside the housing. Right bottom shows the KID chip design holding 56 resonators, each with a size of about 0.9 mm (Color figure online)

We have measured phonon signals from KID resonators as a demonstration with the readout equipment we developed. Figure 3 shows the design of the KID we used for the measurement and the setup of the $\alpha$ particle irradiations. The KID consists of 56 resonators made of aluminum with a thickness of 100 nm formed on a $10\times 10$ mm$^2$ silicon substrate. Each resonator has a size of about $0.9\times 0.8$ mm$^2$. The KID was fabricated using a clean room facility at KEK. We placed the KID chip in a cryostat and cooled it down to 0.3 K. The chip was placed in a brass housing which also held an Am-241 source that irradiated 5.5 MeV $\alpha$ particles to the back side of the KID. Using a vector network analyzer, we identified 15 resonances in a frequency range from 4.0 to 4.2 GHz and set the frequency values in the digital system with a tuning of the LO frequency so that the microwave frequency range could cover the 15 resonators.

**Fig. 4:**
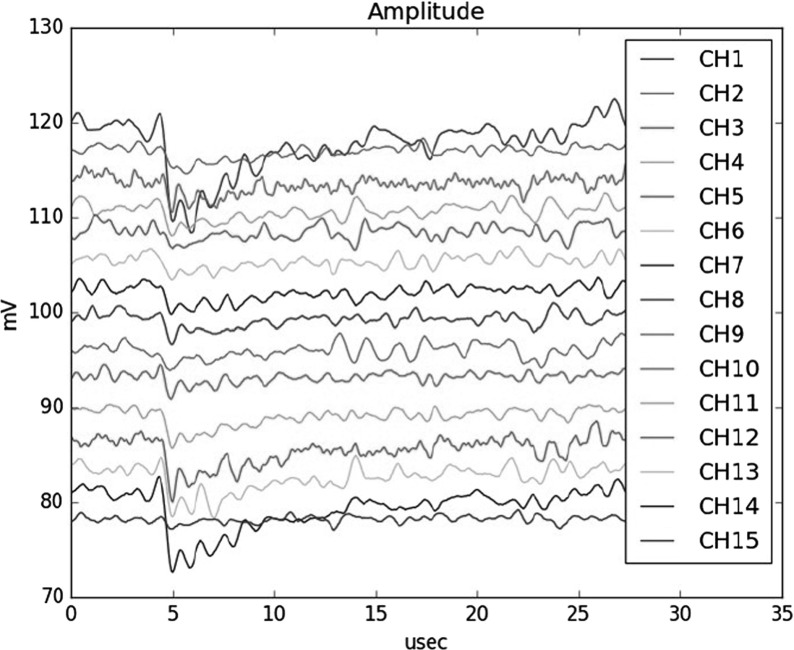
Athermal phonon signals of 15 resonators. The data are read out simultaneously using a single wire with the frequency-domain multiplexing and our readout system (Color figure online)

Figure 4 shows a typical readout result of the 15-channel athermal phonon signals; we successfully read out the KID pulse signals of multiple channels simultaneously in the frequency domain with the self-trigger system. The pulse height difference in individual channels is supposed to be caused by the difference in the total phonon energies deposited to the resonators: The smaller the distance between the resonator and the hit position of an $\alpha$ particle on the substrate, the larger the total energy deposited, as demonstrated by [11]. The trigger rate was measured to be about 10 Hz, which is comparable to the expectations estimated from the radiation source intensity and the detector acceptance. In the measurements, the $\alpha$ particle ray was not focused, and the hit positions of individual $\alpha$ particles were unknown. In order to determine the signal-to-noise ratio, we may need to illuminate the $\alpha$ particle ray to a specific position to reduce the position dependence for the total energy estimation, and then we may identify the deposited energy into individual resonators by computing the fraction [11]. Such study will be conducted as a future subject.

## Summary

We have built a KID readout system using an off-the-shelf FPGA board. The FPGA firmware has been developed to collect pulse signals in the frequency domain by implementing a self-trigger system. We have successfully obtained pulse signal data of 15 resonators simultaneously for athermal phonons produced by irradiating $\alpha$ particles from a sealed $^{241}$Am source to the silicon substrate with an event rate of 10 Hz. This system can be easily extended to read out hundreds to thousands resonators using multiple boards.

## References

[1] Day PK, LeDuc HG, Mazin BA, Vayonakis A, Zmuidzinas J (2003). Nature.

[2] Doyle S, Mauskopf P, Naylon J, Porch A, Duncombe C (2008). J. Low Temp. Phys..

[3] Bourrion O, Benoit A, Bouly JL, Bouvier J, Bosson G, Calvo M, Catalano A, Goupy J, Li C, Macias-Pérez JF, Monfardini A, Tourres D, Ponchant N, Vescovi C (2016). JINST.

[4] van Eyken JC, Strader MJ, Walter AB, Meeker SR, Szypryt P, Stoughton C, O’Brien K, Marsden D, Rice NK, Lin Y, Mazin BA (2015). Astrophys. J. Suppl. Ser..

[5] Mazin BA, Meeker SR, Strader MJ, Szypryt P, Marsden D, van Eyken JC, Duggan GE, Walter AB, Ulbricht G, Johnson M, Bumble B, O’Brien K, Stoughton C (2013). PASP.

[6] https://casper.berkeley.edu/wiki/ROACH

[7] McHugh S, Mazin BA, Serfass B, Meeker S, O’Brien K, Duan R, Raffanti R, Werthimer D (2012). Rev. Sci. Instrum..

[8] Hattori K, Hazumi M, Ishino H, Kibayashi A, Kibe Y, Mima S, Okamura T, Sato N, Tomaru T, Yamada Y, Yoshida M, Yuasa T, Watanabe H (2013). Nucl. Instr. Methods A.

[9] Sakai K, Yamamoto R, Takei Y, Mitsuda K, Yamasaki NY, Hidaka M, Nagasawa S, Kohjiro S, Miyazaki T (2016). J. Low Temp. Phys..

[10] Uchida T, Trans IEEE (2008). Nucl. Sci..

[11] Moore DC, Golwala SR, Bumble B, Cornell B, Day PK, LeDuc HG, Zmuidzinas J (2012). Appl. Phys. Lett..

